# The number of discharge medications predicts thirty-day hospital readmission: a cohort study

**DOI:** 10.1186/s12913-015-0950-9

**Published:** 2015-07-23

**Authors:** David Picker, Kevin Heard, Thomas C. Bailey, Nathan R. Martin, Gina N. LaRossa, Marin H. Kollef

**Affiliations:** Division of Pulmonary & Critical Care Medicine, Washington University School of Medicine, 660 South Euclid Ave., Campus Box 8052, St. Louis, MO 63110 USA; Center for Clinical Excellence, BJC Learning Institute (BLI), 8300 Eager Road, St. Louis, MO 63144 USA; Division of Infectious Diseases, Washington University School of Medicine, 660 South Euclid Ave., Campus Box 8051, St. Louis, MO 63110 USA; Division of Hospital Medicine, Washington University School of Medicine, 660 South Euclid Ave., Campus Box 8058, St. Louis, MO 63110 USA

**Keywords:** Readmission, Discharge medications, Polypharmacy, Outcomes

## Abstract

**Background:**

Hospital readmission occurs often and is difficult to predict. Polypharmacy has been identified as a potential risk factor for hospital readmission. However, the overall impact of the number of discharge medications on hospital readmission is still undefined.

**Methods:**

To determine whether the number of discharge medications is predictive of thirty-day readmission using a retrospective cohort study design performed at Barnes-Jewish Hospital from January 15, 2013 to May 9, 2013. The primary outcome assessed was thirty-day hospital readmission. We also assessed potential predictors of thirty-day readmission to include the number of discharge medications.

**Results:**

The final cohort had 5507 patients of which 1147 (20.8 %) were readmitted within thirty days of their hospital discharge date. The number of discharge medications was significantly greater for patients having a thirty-day readmission compared to those without a thirty-day readmission (7.2 ± 4.1 medications [7.0 medications (4.0 medications, 10.0 medications)] versus 6.0 ± 3.9 medications [6.0 medications (3.0 medications, 9.0 medications)]; *P* < 0.001). There was a statistically significant association between increasing numbers of discharge medications and the prevalence of thirty-day hospital readmission (*P* < 0.001). Multiple logistic regression identified more than six discharge medications to be independently associated with thirty-day readmission (OR, 1.26; 95 % CI, 1.17–1.36; *P* = 0.003). Other independent predictors of thirty-day readmission were: more than one emergency department visit in the previous six months, a minimum hemoglobin value less than or equal to 9 g/dL, presence of congestive heart failure, peripheral vascular disease, cirrhosis, and metastatic cancer. A risk score for thirty-day readmission derived from the logistic regression model had good predictive accuracy (AUROC = 0.661 [95 % CI, 0.643–0.679]).

**Conclusions:**

The number of discharge medications is associated with the prevalence of thirty-day hospital readmission. A risk score, that includes the number of discharge medications, accurately predicts patients at risk for thirty-day readmission. Our findings suggest that relatively simple and accessible parameters can identify patients at high risk for hospital readmission potentially distinguishing such individuals for interventions to minimize readmissions.

## Background

Hospital readmissions within thirty days of hospital discharge occur often and are difficult to predict [1]. In response to the recommendations in the 2007 Medicare Payment Advisory Commission report, many hospitals have dedicated staff efforts and resources in order to identify patients at risk for readmission, as well as to prevent such readmissions [1] (www.medpac.gov/documents/jun07_entirereport.pdf (Accessed 27 June 2014)). The recommendations from the 2007 Medicare Payment Advisory Commission became the basis for the Hospital Readmissions Reduction Program in the Affordable Care Act altering the criteria for hospital payment reimbursement. The Centers for Medicare & Medicaid Services has been charged with enforcement of these criteria by reducing Medicare payments to hospitals that exceed preset all-cause readmission rates [1]. For fiscal year 2013 the readmission penalties have been applied to three conditions: acute myocardial infarction, heart failure, and pneumonia. By 2015, the policy is expected to include readmissions for chronic obstructive pulmonary disease, coronary artery bypass graft surgery, percutaneous coronary interventions, and other vascular procedures [2]. In fiscal year 2013, the maximum payment reduction for hospitals with excess ratios of actual to expected readmission rates was 1 % but increases to 2 % in 2014, and to 3 % for 2015 and beyond [3].

Interventions aimed at preventing readmissions have not been universally successful [4]. One potential explanation for this is the inability to reliably predict which patients are at risk for readmission in order to target readmission prevention interventions. Predictors of hospital readmission can be disease specific such as presence of multivessel disease in patients hospitalized with myocardial infarction [5] or more general such as gender and lack of available follow-up post discharge [6]. Polypharmacy has been identified as a risk factor for readmission for patients discharged from internal medicine services [7]. However, the overall impact of the number of discharge medications on hospital readmission is still undefined. Therefore, we performed a study to examine the influence of the number of discharge medications on the prevalence of thirty-day readmission.

## Methods

### Study location

The study was conducted on eight adult inpatient medicine units of Barnes-Jewish Hospital, a 1250-bed academic medical center in St. Louis, MO (January 15, 2013 to May 9, 2013). Patient care on the inpatient medicine units is delivered by either attending hospitalist physicians or house staff physicians under the supervision of an attending physician. The study was approved by the Washington University School of Medicine Human Studies Committee and informed consent was waived.

### Study overview

We retrospectively evaluated all subjects admitted to one of the eight adult inpatient medicine units of Barnes-Jewish Hospital. The study only included adult patients (aged >18 years) admitted through the emergency department or transferred directly to the hospital from other institutions. We further excluded patients who died while hospitalized as they are necessarily not at risk for readmission. All data was derived from the hospital informatics database provided by the Center for Clinical Excellence, BJC HealthCare. Medications are provided to patients at the time of hospital discharge either through the hospital pharmacy in the form of a thirty-day supply or via a physician authorized prescription to be filled at a local pharmacy.

### Primary end point

Readmission for any reason (i.e., all-cause readmission) to an acute care facility in the thirty days following discharge after the initial hospitalization served as the primary end point. The index hospital serves as the main teaching institution for BJC Healthcare, a large integrated healthcare system of both inpatient and outpatient care. The system includes a total of twelve hospitals in a compact geographic region surrounding and including St Louis, Missouri, and we included readmission to any of these hospitals in our analysis. Persons treated within this healthcare system are, in nearly all cases, readmitted to one of the system’s participating twelve hospitals. If a patient who receives healthcare in the system presents to a nonsystem hospital, he/she is often transferred back into the integrated system because of issues of insurance coverage. Patients with a thirty-day readmission were compared to those without a thirty-day readmission.

### Definitions and variables

Discharge medications were defined as all medications prescribed by the patient’s treating physicians that were required to be taken by the patient at the time of hospital discharge. Discharge medications only included medications needing to be taken on a regular basis, did not include any alternative non-prescribed medications such as herbal or nutritional supplements, and did not include over the counter medications that patients could purchase on their own without a physicians’ prescription. Beyond the number of discharge medications, we recorded information regarding demographics, median income of the zip code of residence as a marker of socioeconomic status, admission to any BJC Healthcare facility within six months of the index admission, comorbidities, and process of care variables. To assess a subject’s chronic health state, we examined if they suffered from coronary artery disease, chronic obstructive pulmonary disease, diabetes mellitus, dementia, had a history of a stroke, and underlying malignancy. To represent the global burden of comorbidities in each patient, we calculated their Charlson scores [8].

### Statistical analysis

The number of patients admitted to the eight adult inpatient medicine units of Barnes-Jewish Hospital determined the sample size. We used the Fisher exact test or Student *t* test, as appropriate, for univariate analyses. The Mann–Whitney *U* test was used for continuous, nonparametrically distributed data. All analyses were 2-tailed, and a *P* value of < 0.05 was assumed to represent statistical significance. We relied on logistic regression for identifying variables independently associated with thirty-day readmission. Based on univariate analysis, variables significant at the *P* < 0.10 level were entered into the model. To arrive at the most parsimonious model, we utilized a stepwise backward elimination approach. We evaluated colinearity with correlation matrices. We report adjusted odds ratios (AORs) and 95 % confidence intervals (CIs) where appropriate. The model’s goodness-of-fit was assessed via calculation of the *R*^2^ value. We conducted a cross-validation of the model in order to assess for overfitting. We reran the logistic model on 90 % of the sample sequentially dropping 10 % of the population with each run. We contrasted the mean accuracy of these analyses with the overall accuracy of the model developed with the entire cohort. Cut-points of variables were determined based on receiver-operating characteristic (ROC) curves. All analyses were performed with SPSS software, version 19.0 (IBM SPSS, Chicago, Illinois).

## Results

The final cohort had 5507 patients with a mean age of 56.1 ± 18.1 years and 2643 (48.0 %) males. The most common reasons for hospital admission were sepsis syndrome including pneumonia and urinary tract infections (21.4 %), congestive heart failure (16.1 %), respiratory distress including chronic obstructive pulmonary disease (13.4 %), acute or chronic renal failure (9.3 %), gastrointestinal disorders (8.8 %), and diabetes mellitus management (7.3 %). Overall, there were 1147 (20.8 %) patients who were readmitted within thirty days of their hospital discharge date.

**Fig. 1:**
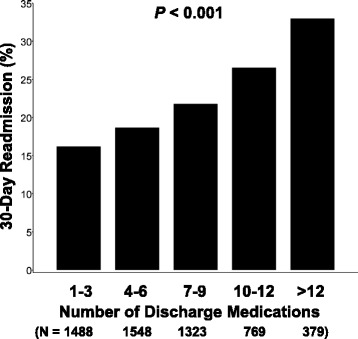
Prevalence of thirty-day hospital readmission as a function of the number of discharge medications

The number of discharge medications was significantly greater for patients having a thirty-day readmission compared to those without a thirty-day readmission (7.2 ± 4.1 medications [7.0 medications (4.0 medications, 10.0 medications)] versus 6.0 ± 3.9 medications [6.0 medications (3.0 medications, 9.0 medications)]; p < 0.001). Figure 1 demonstrates the distribution of thirty-day readmissions according to the number of discharge medications. There was a statistically significant association between increasing numbers of discharge medications and the prevalence of thirty-day hospital readmission. We also observed a direct correlation between increasing number of discharge medications and emergency department visits in the six months prior to the index hospitalization (Spearman’s coefficient = 0.119, *P* < 0.001).

Table 1 shows the characteristics of patients readmitted within thirty days and of patients not requiring hospital readmission within thirty days. Patients requiring hospital readmission within thirty days were younger and more likely to be African-American compared to patients not readmitted within thirty days. Readmitted patients had significantly more comorbidities as manifested by significantly larger Charlson scores and individual comorbidities including coronary artery disease, congestive heart disease, peripheral vascular disease, chronic obstructive pulmonary disease, connective tissue disease, peptic ulcer disease, cirrhosis, diabetes mellitus, paralysis, renal failure, underlying malignancy, lymphoma, and metastatic cancer. Patients with a thirty-day readmission were more likely to be transferred to the intensive care unit during their hospital stay, had longer durations of hospitalization, more emergency department visits in the six month period prior to their hospitalization, significantly lower minimum hemoglobin measurements during hospitalization, significantly higher maximum serum creatinine values, and were more likely to have Medicaid insurance compared to patients without a thirty-day readmission.

**Table 1:** Baseline Patient Characteristics

Variable	30-day Readmission		*P* Value
	Yes (N = 1147)	No ( N = 4360)	
Age, yrs	55.2 ± 17.7	56.4 ± 18.1	0.044
Gender: Male	545 (47.5)	2098 (48.1)	0.716
Female	602 (52.5)	2262 (51.9)	
Race: Caucasian	523 (45.6)	2167 (49.7)	0.034
African-American	539 (47.0)	1919 (44.0)	
Other	85 (7.4)	273 (6.3)	
Median Income, dollars	30037 [24830,36763]	29281 [25118,37470]	0.631
Number of Discharge Medications	7 [4,10]	6 [3,9]	<0.001
Charlson Comorbidity Score	5 [2,8]	3 [1,6]	<0.001
ICU transfer during admission	171 (14.9)	541 (12.4)	0.025
Myocardial Infarction	203 (17.7)	525 (12.0)	<0.001
Congestive Heart Failure	386 (33.7)	929 (21.3)	<0.001
Peripheral Vascular Disease	207 (18.0)	430 (9.9)	<0.001
Cardiovascular Disease	180 (15.7)	508 (11.7)	<0.001
Dementia	37 (3.2)	101 (2.3)	0.080
Chronic Obstructive Pulmonary Disease	499 (43.5)	1375 (31.5)	<0.001
Connective Tissue Disease	93 (8.1)	222 (5.1)	<0.001
Peptic Ulcer Disease	124 (10.8)	283 (6.5)	<0.001
Cirrhosis	118 (10.3)	208 (4.8)	<0.001
Diabetes Mellitus without complications	260 (22.7)	843 (19.3)	0.012
Diabetes Mellitus with complications	207 (18.0)	433 (9.9)	<0.001
Paralysis	76 (6.6)	173 (4.0)	<0.001
Renal failure	376 (32.8)	873 (20.0)	<0.001
Underlying Malignancy	99 (8.6)	250 (5.7)	<0.001
Lymphoma	29 (2.5)	49 (1.1)	<0.001
Metastatic Cancer	79 (6.9)	184 (4.2)	<0.001
HIV	18 (1.6)	88 (2.0)	0.325
Minimum Hemoglobin, g/dL^a^	9.5 ± 2.3	10.4 ± 2.4	<0.001
Maximum Creatinine, mg/dL^a^	1.16 [0.81,2.14]	1.00 [0.77,1.50]	<0.001
Length of Stay, days	4.1 [2.2,7.4]	3.0 [1.8,5.6]	<0.001
Emergency Department visit in past 6 months	1 [0,3]	0 [0,2]	<0.001
Admitted from Emergency Dept	817 (71.2)	3066 (70.3)	0.548
Insurance: Private	38 (3.3)	251 (5.8)	<0.001
Medicare	184 (16.0)	961 (22.0)	
Medicaid	554 (48.3)	1885 (43.2)	
Patient Pay	49 (4.3)	390 (8.9)	

Values expressed as mean ± standard deviation or median [25th percentile, 75th percentile]

Abbreviations: *ICU* intensive care unit; *HIV* human immunodeficiency virus

^a^During the hospitalization

Table 2 shows the results of a multiple logistic regression model derivation to examine the variables associated with thirty-day hospital readmission in this population. In this model, more than one emergency department visit in the previous six months was the strongest predictor of thirty-day readmission, with an AOR of 2.37 (95 % CI, 2.19 to 2.57; *P* < 0.001). Other independent predictors of thirty-day readmission included more than six discharge medications, a minimum hemoglobin value less than or equal to 9 g/dL, presence of peripheral vascular disease, congestive heart failure, cirrhosis, and metastatic cancer. The model had a good fit of the data with an *R*^2^ of 0.228. Based on cross-validation, we did not observe overfitting. The AORs for the variables that were significant predictors in the model in all of the ten sequential reruns (with 90 % of the cohort) did not differ from the findings in the complete population.

**Table 2:** Variables Independently Associated with Thirty-Day Readmission

Variables	OR	95 % CI	P value	Risk Score
Number of Discharge Medications >6	1.26	1.17–1.36	0.003	1.0
Number of ED visits > 1^ab^	2.37	2.19–2.57	<0.001	2.0
Minimum Hb value ≤ 9 gm/dl	1.67	1.54–1.81	<0.001	1.5
Congestive Heart Failure	1.52	1.40–1.67	<0.001	1.5
Peripheral Vascular Disease	1.42	1.28–1.59	0.001	1.5
Cirrhosis	1.26	1.21–1.32	<0.001	1.0
Metastatic Cancer	1.08	1.06–1.11	0.002	1.0

^a^Within 6 months of the index hospitalization

^b^Cut-points for number of discharge medications, emergency department visits, and minimum Hg value were assessed by receiver-operating characteristic curves as described in the Methods section

Abbrevations: *OR* odds ratio, *CI* confidence interval, *ED* emergency department, *Hb* hemoglobin

**Fig. 2:**
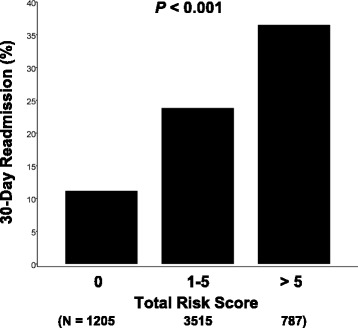
Prevalence of thirty-day hospital readmission as a function of the Risk Score. (See Methods section for a description of how the Risk Score was calculated)

**Fig. 3:**
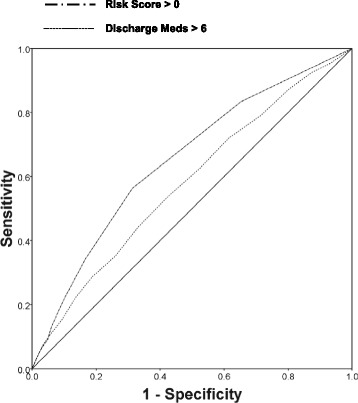
Receiver-operating characteristic curves. Abbreviation: Meds = medications

Risk scores for thirty-day readmission were derived by converting the adjusted odds ratios for the independent predictors of thirty-day readmission identified in the multiple logistic regression analysis to risk scores as shown in Table 2. The maximum possible risk score was a value of 9.5. Figure 2 demonstrates the distribution of calculated risk scores relative to the prevalence of thirty-day hospital readmission. As the scores increased, so did the probability of thirty-day readmission. For example, among patients with a score of zero, the prevalence of thirty-day readmission was approximately eleven percent, while more than thirty-five percent of those with a score greater than five had a thirty-day readmission (*P* < 0.001). Figure 3 reveals the ROC curves for the risk score and the number of discharge medications. As the ROC curves document, the risk score had higher sensitivity than the number of discharge medications for nearly the entire range of specificities. Reflecting this, the area under the ROC (AUROC) curve was greater for the risk score. The AUROC curve for the number of discharge medications equaled 0.579 (95 % CI, 0.560–0.598) whereas the AUROC curve for the risk score was 14.0 % higher (0.661 [95 % CI, 0.643–0.679]; *P* = 0.030 for the difference in AUROC curves).

A comparison of screening characteristics using the number of discharge medications greater than six and a risk score greater than zero for identifying patients with a thirty-day readmission is presented in Table 3. Overall, the risk score proved more accurate having greater sensitivity and specificity compared to the number of discharge medications alone.

**Table 3:** Screening Characteristics of the Number of Discharge Medications Versus Risk Score

Characteristic	Sensitivity	Specificity	PPV	NPV	Accuracy
Number of Discharge Medications >6	53.8	57.5	25.0	82.5	56.7
Risk Score > 0	83.4	65.4	25.1	93.8	69.1

Abbreviations: *PPV* positive predictive value; *NPV* negative predictive value

## Discussion

The main finding of this study was that the number of medications at the time of hospital discharge was associated with unplanned rehospitalization within thirty days in patients discharged from internal medicine units. However, the overall predictive accuracy for thirty-day readmission was found to be significantly better for a risk score that incorporated the number of discharge medications as one of the predictive elements, compared to using the number of discharge medications as a sole predictor. We also identified other predictors of thirty-day readmission including the number of emergency department visits in the previous six months, the minimum hemoglobin value during hospitalization, presence of congestive heart failure, peripheral vascular disease, cirrhosis, and metastatic cancer.

Our results are consistent with those recently published from other settings. Wimmer et al. examined a small cohort of patients discharged from a geriatric unit in Australia and found that medication regimen complexity was not associated with unplanned hospital readmission in older people [9]. However, in patients discharged to nonhome settings, the number of discharge medications predicted rehospitalization suggesting that a patient’s discharge destination may be an important factor in unplanned medication-related readmissions [9]. A recent systematic review also found that hospitalization resulting from medication related problems was common with the main causes being adverse drug reactions and medication non-compliance [10]. This review also found that old age and polypharmacy were highly represented among patients admitted to hospitals due to medication related problems [10]. Our study further highlights the nature and extent of medication-related hospitalizations in several ways. First, increasing medication use is likely a surrogate marker of disease severity and complexity making it a good marker for readmission risk. Secondly, as the number of medications increases it is more likely that patients will not be compliant with all prescribed medications due to either cost issues, side effects, or inability to keep accurate tracking of all their medication consumption.

Quality improvement programs have been implemented to reduce the number and complexity of discharge medications in order to minimize adverse events and to improve compliance. A pharmacist-directed discharge medication management program has been shown to be successful in decreasing both number and type of discharge medications [11]. Overall, patient system readmission rates were also significantly decreased in association with implementation of the discharge medication management program [11]. However, other programs aimed at minimizing problems with discharge medications to include education and medication reconciliation have not been able to show reductions in the occurrence of hospital readmission [12, 13]. These studies would suggest that the intricacy of discharge medications, to include their overall number, may simply reflect the global medical complexity of patients which is the more important overall determinant of subsequent readmission [14].

The difficulty of understanding the determinants of readmission and reducing readmission events is illustrated by several recent studies. Kangovi et al. performed a randomized trial at two hospitals randomizing usual care versus creating individualized action plans for achieving patients’ stated goals for recovery prior to discharge administered by community health workers [15]. Although medication adherence was not influenced by this intervention, timely posthospital primary care and high-quality discharge communication occurred resulting in fewer multiple thirty-day readmissions. Similarly, Hochhalter et al. carried out a retrospective study to test the association of long-term medication adherence with hospital readmission in a cohort of beneficiaries enrolled in a Medicare Cost Plan [16]. They found that medication adherence was not associated with thirty-day readmission, but older age and longer length of hospital stay were associated with higher likelihood of thirty-day readmission, while having an office visit within thirty days of discharge was associated with lower odds of readmission [16]. Dharmarajan et al. showed in a large database that readmissions following congestive heart failure, acute myocardial infarction, and pneumonia hospitalization were diverse and usually for a different reason than the original hospitalization problem [14]. Taken together, these studies illustrate the complexity of identifying specific interventions, such as improved medication adherence, in order to reduce readmissions. These studies also suggest that more general interventions such as early posthospital follow up visits may be more important means of preventing readmissions.

Our study has several important limitations. First, this was a retrospective analysis. It is possible that we did not identify all patient variables and processes of care that are important determinants of thirty-day readmission in this population. Moreover, the retrospective nature of this investigation did not allow an assessment of medication adherence as it relates to the number of discharge medications. Second, we simply examined the number of discharge medications without determining whether certain classes of discharge medications were more likely to be associated with thirty-day readmission. Third, we did not have access to outpatient visits following the index hospitalization. Several studies have identified timely posthospital discharge as an important process for the prevention of readmissions [15, 16]. Fourth, this was a single-center study in a medical population, which limits the generalizability of our findings. Additionally, the patient population at Barnes-Jewish Hospital may not be representative of that at other centers. For example, the mean age of our population is younger than that reported in other studies examining risk factors for hospital readmission [16]. However, a strength of our study is that we did not limit inclusion to patients with specific diseases. We also did not examine other potentially important predictors of hospital readmission to include the presence of delirium. Finally, we did not account for patients who died within thirty days after hospital discharge. These represent discharged patients that could not be readmitted but who may have shared characteristics of the patients at high risk for readmission. However, we found that the number of discharge medications was associated with the prevalence of thirty-day readmission despite this bias adding to the robustness of our findings.

## Conclusions

The number of discharge medications was found to be associated with the prevalence of thirty-day hospital readmission. A risk score that includes the number of discharge medications accurately predicts patients at risk for thirty-day readmission. Our findings suggest that relatively simple and accessible parameters can identify patients at high risk for hospital readmission potentially distinguishing such individuals for interventions to minimize readmissions.

## Abbreviations

AOR: Adjusted odds ratio
AUROC: Area under Receiver Operating Characteristic
CI: Confidence interval
ROC: Receiver-operating characteristic
